# *e*Volver: an optimization engine for evolving protein sequences to stabilize the respective structures

**DOI:** 10.1186/1756-0500-6-303

**Published:** 2013-07-31

**Authors:** Michal Brylinski

**Affiliations:** 1 Department of Biological Sciences, Louisiana State University, Baton Rouge, LA 70803, USA; 2 Center for Computation & Technology, Louisiana State University, Baton Rouge, LA 70803, USA

**Keywords:** Artificial sequences, Evolved sequences, Protein threading, Homology searches, Protein structure modeling, Template-based modeling

## Abstract

**Background:**

Many structural bioinformatics approaches employ sequence profile-based threading techniques. To improve fold recognition rates, homology searching may include artificially evolved amino acid sequences, which were demonstrated to enhance the sensitivity of protein threading in targeting midnight zone templates.

**Findings:**

We describe implementation details of *e*Volver, an optimization algorithm that evolves protein sequences to stabilize the respective structures by a variety of potentials, which are compatible with those commonly used in protein threading. In a case study focusing on LARG PDZ domain, we show that artificially evolved sequences have quite high capabilities to recognize the correct protein structures using standard sequence profile-based fold recognition.

**Conclusions:**

Computationally design protein sequences can be incorporated in existing sequence profile-based threading approaches to increase their sensitivity. They also provide a desired linkage between protein structure and function in *in silico* experiments that relate to e.g. the completeness of protein structure space, the origin of folds and protein universe. *e*Volver is freely available as a user-friendly webserver and a well-documented stand-alone software distribution at http://www.brylinski.org/evolver.

## Background

In template-based protein structure modeling, sequence profile-based threading and fold recognition approaches [1] frequently fail to detect in the Protein Data Bank (PDB) [2] structurally similar templates whose sequence similarity to the target falls into the midnight zone [3]. This is due to the fact that the vast majority of midnight zone pairs of proteins with similar structures are likely the products of convergent or divergent evolution [4,5]. To address this problem, computationally designed protein sequences have been proposed to support fold recognition and homology searching [6-8]. Recently, we demonstrated that using synthetic sequences artificially evolved for the template structures rather than (or in addition to) wild-type sequences indeed improves fold recognition rates [9]. These synthetic sequences provide an orthogonal source of signal that could be advantageously exploited in protein structure modeling. Here, the critical component is an efficient engine that optimizes amino acid sequences to stabilize the respective structures. It needs to be effective, consistent with scoring functions used in threading and fold recognition and devoid of potential modeling artifacts, such as the grouping of a particular type of residues.

In this communication, we describe recently developed software, *e*Volver, which optimizes protein-like amino acid sequences to stabilize the respective structures. In previous large-scale benchmarks, it was shown to generate synthetic sequences, which despite their low (14% on average) identity to the wild-type sequences have significant capabilities to recognize native-like folds [9]. Here, we focus on the details of software implementation and usage, perform computational resource profiling, and discuss a case study using leukemia-associated RhoGEF (LARG).

## Findings

### Scoring function for the evolution of synthetic sequences

The force field used by *e*Volver for sequence optimization combines several energy terms: a burial potential, secondary structure preferences, a distant-dependent contact potential, sequence profiles and anti-grouping restraints, described in detail in [9]. The burial potential uses a 7-state alphabet, BURIAL-Cβ-14-7, which arranges protein residues according to their exposure to solvent and neighboring atoms [10]. Secondary structure preferences were derived from the non-redundant CATH library [11] using a 7-state classification by STRIDE [12]. As a distant-dependent statistical potential, *e*Volver employs a protein conformation free energy score by dFire [13], separately for Cα atoms and the side chain centers of mass. The original dFire pseudo-energies are linearly transformed to make these scores independent of protein length. For a given target structure, sequence profiles are derived from statistically significant (at a TM-score of ≥0.4 [14]) structure alignments constructed by Fr-TM-align [15] against either CATH [11] (domain library) or PDB [2] (chain library). To improve the signal to noise ratio in low-homology sequence profiles, we use a 7-state residue classification by amino acid type (small polar, large polar, negatively charged, positively charged, hydrophobic, aromatic and histidine) [16]. Finally, grouping artifacts are suppressed by Helmut Schmidt's test of force-like runs, also known as the Pot statistics [17]. This scoring term penalizes the artificial short-range clustering of particular amino acid types. Our initial tests showed that using a scoring function lacking anti-grouping restraints frequently leads to α-helices overpopulated with clusters of alanine residues and β-structures mainly composed of groups of isoleucine and valine residues. The combined scoring function consists of a linear combination of weighted pseudo-energy terms. To maximize the accuracy, the weight factors were optimized on a large dataset of native-like and decoy sequences constructed for the CATH library [11].

### Sequence optimization engine

Simulated Annealing Monte Carlo (SA) is a random search technique, which exploits an analogy between statistical mechanics of a metal cooling and freezing into a minimum energy crystalline state and finding the minimum of a multivariate function in general optimization problems [18]. To efficiently explore the target sequence space, *e*Volver uses a fast implementation of SA from GNU Scientific Library [19]. The following cooling scheme and SA parameters are used: N_TRIES = 200 (number of tries before stepping), ITERS_FIXED_T = 2000 (number of iterations for each temperature), K = 1.0 (Boltzmann constant), T_INITIAL = 5000 (initial temperature), MU_T = 1.002 (damping factor for temperature) and T_MIN = 0.005 (final temperature).

An important component of an SA code is the random number generator, which is used to introduce random perturbations in the control variables as well as to calculate the Metropolis-Hastings acceptance criterion [20]. A typical SA simulation by *e*Volver comprises >1.3×10^7^ iterations, therefore the random number generator used should have good spectral properties (a mathematical measurement of randomness). *e*Volver employs a high-quality random number generator MT19937, which is a variant of the twisted generalized feedback shift-register algorithm, also known as the “Mersenne Twister” generator [21]. The default seed used in *e*Volver reproduces the original generator, which has passed the Diehard suite of statistical tests for assessing the randomness quality [22]. Moreover, the results are fully reproducible across different operating systems and hardware architectures. As a consequence of this high reproducibility, multiple runs are not needed for a given initial sequence.

### Input files for *e*Volver and output data

*e*Volver requires three input files: a single-chain target structure in PDB format, a secondary structure assignment by STRIDE [12], and a structure-based sequence profile. The latter can be generated from structural analogs by eprofile, a tool included in the *e*Volver software distribution. The original benchmarking results for *e*Volver were obtained using sequence profiles generated by Fr-TM-align [15] at a TM-score threshold of ≥0.4 [9]. However, any other statistically validated structure alignment program can be used instead, e.g. CE [23], MAMMOTH [24], DALI [25], etc. We note that the webserver requires only a target structure; the remaining files are generated automatically. Moreover, two non-redundant structure libraries are currently available for the construction of sequence profiles: CATH [11] (domain library) and PDB [2] (chain library). In general, the former should be used for single-domain targets, whereas the latter can result in more sensitive sequence profiles for multiple-domain targets.

SA simulations can start from either a native sequence read from the input PDB file, a shuffled native sequence that preserves the native sequence composition, or a random protein-like sequence. The first two options are useful in benchmarking calculations, whereas random initial sequences, which are generated according to amino acid frequencies provided by UniProtKB/Swiss-Prot [26], are a good choice for real applications.

**Figure 1:**
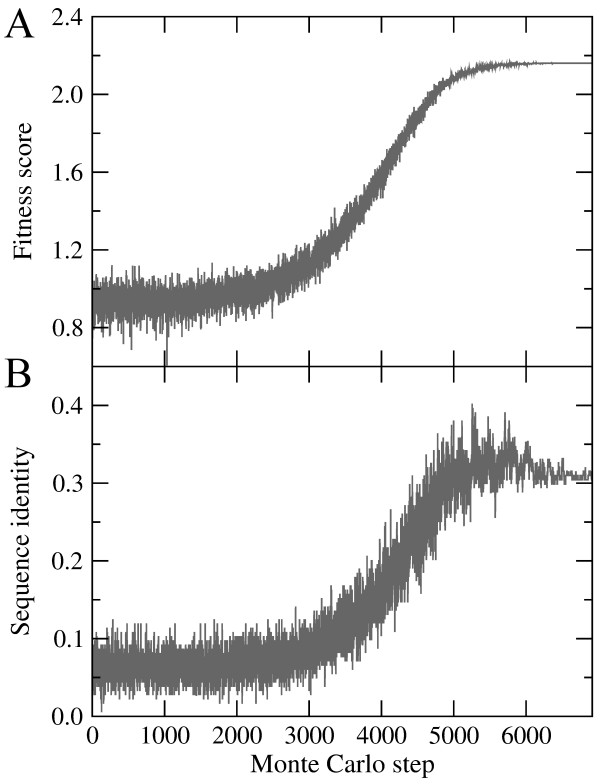
**Simulated Annealing trajectories generated by *e*Volver for topoisomerase II domain 5. (A)** Fitness score and **(B)** the identity of evolving sequence to the native sequence are plotted as a function of Monte Carlo step.

The main output from *e*Volver is a sequence artificially evolved to stabilize the target structure in our force field. In addition, generated SA trajectories can be used to visualize the progress of the simulated sequence evolution. In Figure 1, we analyze a trajectory obtained for topoisomerase II domain 5 from *Saccharomyces cerevisiae* S288c [27] (PDB-ID: 3l4jA03, CATH classification: 3.90.199.10). At initial high-temperature stages of this simulation, the fitness score fluctuates around a value of 1.0, which corresponds to a random score (Figure 1A). Cooling the system down gradually decreases the acceptance ratio and, consequently, increases the overall fitness score. At the end of simulation, the evolving sequence is “frozen” into a maximum fitness of 2.16, which is likely the global pseudo-energy minimum state. We note that SA does not guarantee the success in finding a globally optimal solution. Figure 1B shows that the fitness score of evolving sequences is also well correlated with their identities to the native sequence. Random sequences generated at high temperatures have a sequence identity to native of ~8%, which continuously increases and reaches 30% for the final evolved sequence.

### Profiling of computational resources

**Figure 2:**
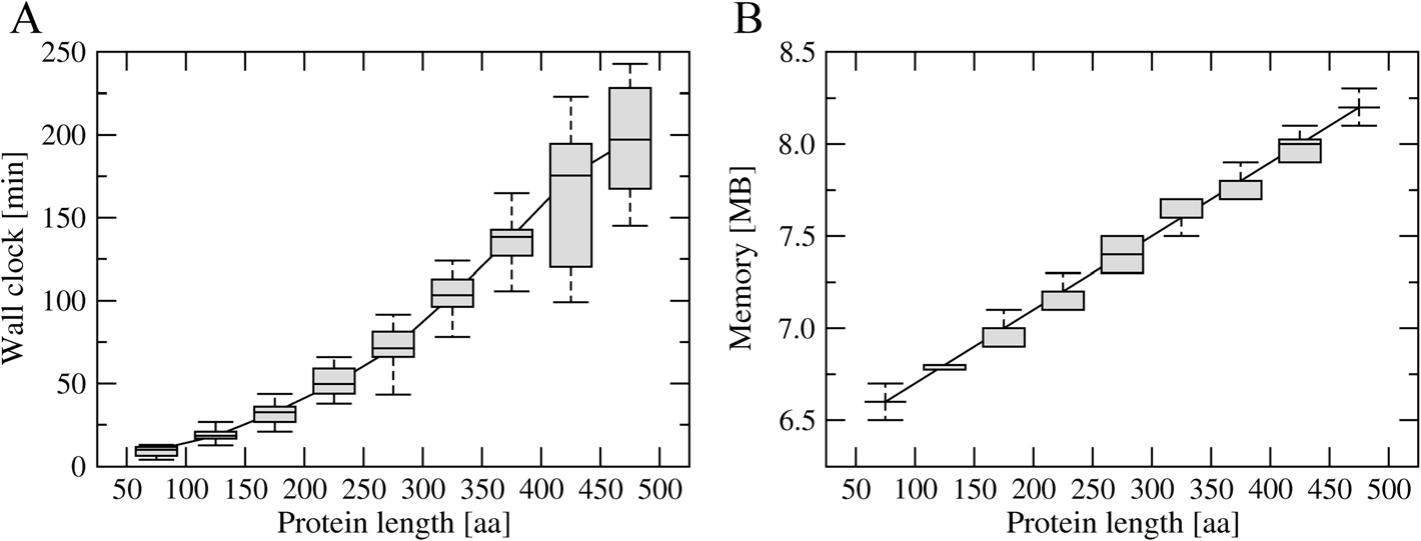
**Utilization of computing resources by *e*Volver.** Average ± standard deviation **(A)** wall clock and **(B)** memory is plotted as a function of the target protein length. Boxes end at the quartiles Q_1_ and Q_3_; a horizontal line in a box is the median. Whiskers point at the farthest points that are within 3/2 times the interquartile range.

Particularly for large-scale applications of *e*Volver, it is essential to estimate the resources needed for individual calculations with respect to the CPU time and memory utilization. The resource profiling is performed on a dataset of 180 proteins randomly chosen from the original *e*Volver benchmarking dataset [9]. These proteins were selected to uniformly populate 9 bins with 20 structures in each bin; the bins evenly span the range of the target sequence length between 50 and 500 residues. The testing system is HP ProLiant SL250s Gen8, which has 2 Intel Xeon E5-2670 8-core processors running at 2.6GHz and it is equipped with 64GB of memory. Figure 2 shows the average ± standard deviation wall clock and memory usage. For proteins shorter than 250 residues in length, *e*Volver typically completes within 1 hour, whereas proteins longer than 400 residues require up to 3 hours of CPU time. Furthermore, the memory consumption by *e*Volver is only 6-8 MB, which scales linearly with the target protein length. This very small memory footprint is particularly appealing for targeting cost-effective accelerators, such as Graphics Processing Units (GPU) or Intel Many Integrated Cores (MIC). In the future, we will develop a parallel version of *e*Volver that can be deployed on heterogeneous high-performance computing systems equipped with accelerator cards.

### Case study

**Figure 3:**
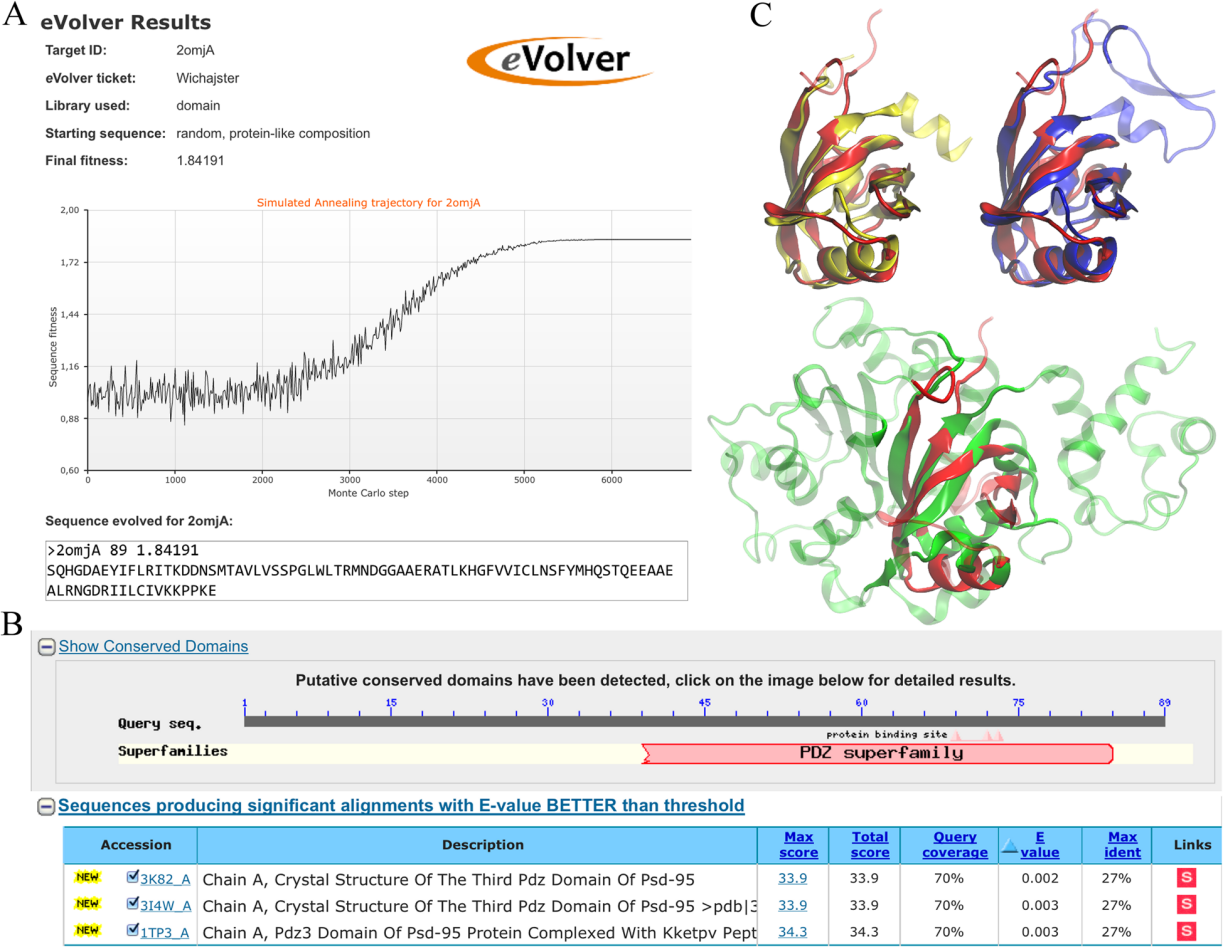
**Synthetic sequence evolved to stabilize the PDZ domain. (A)** Snapshot of the results from *e*Volver webserver, which shows the final fitness score, the SA trajectory and the evolved sequence in FASTA format. **(B)** Output from PSI-BLAST obtained by using the evolved sequence to query PDB. **(C)** Structure alignments of the top 3 PSI-BLAST hits (3k82A – yellow, 3i4wA – green, 1tp3A – blue) against the target structure (2omjA – red); aligned regions are solid.

As a proof of concept, we use *e*Volver to optimize a sequence that stabilizes the structure of the PDZ domain of Rho guanine nucleotide exchange factor 12 (LARG, PDB-ID: 2omjA). Figure 3A presents a snapshot of the results page from the *e*Volver webserver, which shows the optimized sequence evolved from a random protein-like sequence as well as the SA trajectory. Next, we additionally verify the quasi-stability of this evolved sequence by using PSI-BLAST [28] to find in the PDB [2] these proteins that produce significant alignments with E-values <0.005. The results from PSI-BLAST presented in Figure 3B show that the synthetic sequence was correctly assigned to the PDZ superfamily. Furthermore, PSI-BLAST picked out 3 proteins from PDB that produce significant alignments with the evolved sequence: 3k82A, 3i4wA and 1tp3A. All three structures contain a PDZ domain. Structure alignments of these proteins against 2omjA (Figure 3C) result in a TM-score [14] (Cα-RMSD) of 0.74 (2.02 Å), 0.39 (4.48 Å) and 0.73 (2.28 Å), respectively. 3k82A and 1tp3A produce highly significant structure alignment with a TM-score of >0.7, whilst 1i4wA is at the TM-score significance threshold. Note that these proteins were identified using the artificially evolved sequence, which was optimized to stabilize the structure of 2omjA and share only 22% identity with the wild type sequence. Yet, this sequence carries sufficient amount of information to properly recognize structural analogs in the PDB.

## Conclusions

We developed *e*Volver, a method for the optimization of generic protein-like amino acid sequences to stabilize the respective structures. An interesting, and potentially useful in practical applications, feature of these artificially evolved sequences is their high capability to recognize the correct protein structures using standard sequence profile-based approaches to fold recognition. *e*Volver is available as a user-friendly webserver as well as a stand-alone software distribution, which can be installed locally in a high-performance computing environment to optimize amino acid sequences for large datasets, e.g. template libraries or synthetic structures. The former can be used to develop more sensitive threading approaches; the latter are widely used in studies on the completeness of protein structure space [29] as well as in research focusing on the origin of folds and protein universe [30,31]. The effective procedure for the design of a quasi-stable sequence for an arbitrary structure also provides a desired linkage between protein structure and function in computer experiments. This opens up areas for further exploration, which mostly relate to protein evolution, engineering and design as well as the origins of biochemical function.

## Availability and requirements

Project name: *e*Volver

Project home page: http://www.brylinski.org/evolver

Operating system(s): Linux

Programming language: C++

Other requirements: GNU Scientific Library (GSL)

License: GNU GPL

Restrictions to use by non-academics: none

## Competing interests

The author declares that he has no competing interests.
